# Simultaneous idiopathic bilateral sudden hearing loss – characteristics and response to treatment^☆^

**DOI:** 10.1016/j.bjorl.2016.12.003

**Published:** 2017-01-24

**Authors:** Ferit Akil, Umur Yollu, Mehmet Yilmaz, H. Murat Yener, Marlen Mamanov, Ender Inci

**Affiliations:** a Diyarbakir Selahaddin Eyyubi Public Hospital, Otolaryngology Clinic, Diyarbakir, Turkey; b Gumushane Public Hospital, Otolaryngology Clinic, Gumushane, Turkey; c Istanbul University, Cerrahpasa Medical School, Department of Otolaryngology, Istanbul, Turkey

**Keywords:** Sudden hearing loss, Autoimmune diseases, Tinnitus, Treatment, Prognosis, Perda auditiva súbita, Doenças autoimunes, Zumbido, Tratamento, Prognóstico

## Abstract

**Introduction:**

The aetiology of sudden hearing loss is poorly defined; however, infectious, vascular and neoplastic aetiologies are presumed to be responsible. In addition, the aetiology of bilateral sudden hearing loss is also unknown.

**Objective:**

The objective of this study is identify the characteristics and treatment response of simultaneous bilateral sudden hearing loss.

**Methods:**

This is a case–control study that practised in tertiary care academic centre. 132 patients with sudden hearing loss who were treated with systemic steroid and hyperbaric oxygen together were included. 26 patients had bilateral sudden hearing loss and 106 patients had unilateral sudden hearing loss. Patients were evaluated with clinical, audiological and radiological examinations and laboratory tests were done. Findings and response to treatment of the patients were compared.

**Results:**

The mean ages of patients with unilateral and bilateral sudden hearing loss were 42.0 years and 24.5 years respectively with a statistically significant difference (*p* < 0.001). Immune response markers were more prevalent in bilateral sudden hearing loss. Pre-treatment audiologic thresholds were 69.1 dB for unilateral sudden hearing loss and 63.3 dB for the left ears and 67.6 dB for the right ears for bilateral sudden hearing loss without significant difference. Post-treatment average hearing threshold in unilateral sudden hearing loss was 47.0 dB and 55.4 dB for the left ears and 59.0 for the right ears in bilateral sudden hearing loss. Average hearing improvement in unilateral sudden hearing loss group was significant (*p* < 0.001) in spite of it was not significant in bilateral sudden hearing loss group for both ears. Between the groups; there was a significant difference for hearing improvement favouring unilateral sudden hearing loss (*p* < 0.001). Tinnitus scores decreased significantly in both groups of patients (*p* < 0.001) in spite of there was no significant difference between the groups of patients.

**Conclusion:**

Patients with bilateral sudden hearing loss showed lower age, worse prognosis and higher rate of positive immune response markers. Cardiovascular risk factors seem to have an important role in the aetiology of unilateral cases whereas this importance was not present in bilateral ones.

## Resumo

**Introdução:**

A etiologia da perda auditiva súbita ainda não está bem definida; contudo, presume-se que as etiologias infecciosas, vasculares e neoplásicas sejam as responsáveis. Além disso, a etiologia da perda auditiva súbita bilateral também é desconhecida.

**Objetivo:**

O objetivo desse estudo foi identificar as características e a resposta ao tratamento de perda auditiva súbita bilateral simultânea.

**Método:**

Este é um estudo de caso-controle realizado em um centro de atenção terciária. Foram incluídos 132 pacientes com Perda Auditiva Neurossensorial Súbita (PANS) que foram tratados com esteroides sistêmicos e oxigênio hiperbárico. 26 pacientes tiveram PANS bilateral e 106 pacientes tiveram PANS unilateral. Os pacientes foram avaliados com exames clínicos, audiológicos, radiológicos e exames laboratoriais. Os achados e a resposta ao tratamento dos pacientes foram comparados.

**Resultados:**

As idades médias dos pacientes com PANS unilateral e bilateral foram 42,0 anos e 24,5 anos, respectivamente, com diferença estatisticamente significante (*p* < 0,001). Os marcadores de resposta imune foram mais prevalentes na PANS bilateral. Os limiares audiológicos pré-tratamento foram 69,1 dB para PANS unilateral e 63,3 dB para a orelha esquerda e 67,6 dB para a orelha direita para PANS bilateral, sem diferença significativa. O limiar médio de audição pós-tratamento em PANS unilateral foi de 47,0 dB e 55,4 dB para a orelha esquerda e 59,0 para a orelha direita em PANS bilateral. A melhora auditiva média no grupo com PANS unilateral foi significativa (*p* < 0,001), apesar de não ser significativa no grupo com PANS bilateral para ambas as orelhas. Houve diferença significativa entre os grupos na melhora auditiva favorecendo a PANS unilateral (*p* < 0,001). Os escores de zumbido diminuíram significativamente em ambos os grupos de pacientes (*p* < 0,001), apesar de não ter havido diferença significativa entre os grupos de pacientes.

**Conclusão:**

Os pacientes com PANS bilateral eram mais jovens, tinham pior prognóstico e maior taxa de marcadores de resposta imunológica positiva. Os fatores de risco cardiovasculares parecem ter um papel importante na etiologia dos casos unilaterais, ao passo que essa importância não estava presente nos casos bilaterais.

## Introduction

Sudden hearing loss (SHL) is defined as a sensorineural type of hearing loss that develops over a period of 72 h, with 30 dB or more of hearing reduction in at least 3 contiguous frequencies. The severity of the hearing loss varies from patient to patient. SHL can affect any age group, but usually peaks at 60 years of age, without a gender difference.^1^ Ear fullness and tinnitus are common complaints in the affected ear, while varying degrees of vertigo can be detected in 40% of SHL patients.^2^ The estimated incidence of SHL in the United States per year ranges from 5 to 20 cases per 100,000 individuals.^3^ Most cases of SHL develop unilaterally, with bilateral involvement comprising only 0.44–4.9% of the patients,^1^, ^2^, ^3^, ^4^, ^5^ which makes the incidence of bilateral SHL extremely low.^5^ Although rare, this condition is more dramatic due to the bilateral loss of hearing.

The aetiology of sudden hearing loss is poorly defined; however, infectious, vascular and neoplastic aetiologies are presumed to be responsible.^2^, ^3^ In addition, the aetiology of bilateral SHL is also unknown. The presumed factors, such as viral infection, circulatory insufficiency or labyrinthine membrane rupture, are similar to the causes of the unilateral form.^1^, ^2^, ^3^, ^4^, ^5^ Many treatment protocols have been described, but there is no consensus about the treatment modality of choice in SHL. The most widely accepted therapy is tapered oral corticosteroid therapy.^6^ However, a better treatment response has been reported in the literature with the addition of hyperbaric oxygen (HBO) to oral steroid therapy.^7^ Certain prognostic factors can be used in the prediction of the treatment results in unilateral SHL. Overall, the prognosis of SHL is related to age, the interval between the development of the condition and treatment, the presence or absence of vertigo and tinnitus, the degree of hearing loss, and the patterns of the audiogram.^8^ In the cases of bilateral SHL, since the incidence is low, the prognostic factors are not as clear.

In this study, we conducted a retrospective analysis of SHL cases that presented to our clinic. Those patients with simultaneous bilateral SHL were compared to the patients with unilateral loss with respect to the etiological factors, demographics and responses to treatment, in order to better understand this rare condition.

## Methods

Between 2000 and 2012, the charts of 857 patients admitted to the tertiary care academic centre with complaints of SHL were reviewed. In total, 132 patients treated with the same protocol (systemic steroids with HBO treatment), and who met the following criteria were included in this study: no history of previous ear diseases, head injuries and acoustic trauma. SHL was defined as sensorineural hearing loss of at least 30 dB in 3 contiguous frequencies over a period of 3 days or less. The age, sex, duration of symptoms, laterality of the disease, presence of vertigo or dizziness, and cardiovascular risks of the patients were noted. Diabetes mellitus, hypertension and vasculitis were regarded as cardiovascular risks.

Twenty-six patients (16 males, 10 females, mean age 24.5 ± 19.8 years old, range 7–57 years) had bilateral SHL, and 106 patients (56 males, 50 females, mean age 42.0 ± 15.0 years old, range 12–58 years) had unilateral SHL. Each patient was evaluated with a detailed medical history, complete head and neck examination, laboratory tests, serial pure tone audiogram, MRI and CT scan. Blood samples were collected from the patients, a complete blood count and erythrocyte sedimentation rate (ESR) were conducted, and the alanine transaminase (ALT), aspartate transaminase (AST), serum glucose, cholesterol, lipid, urea, creatinine, thyroid hormone and antinuclear antibody (ANA) levels were measured. HEAR staging was used to classify the audiogram characteristics. According to this staging system, while type 0 is flat, central or upward sloping, type 1 is down sloping or indicative of total hearing loss.^1^ We used the Turkish version of the validated questionnaire of the Tinnitus Handicap Inventory (THI) to grade the tinnitus.^9^

Each patient was treated with systemic steroids and HBO. The oral steroid protocol for sudden hearing loss used in our clinic consisted of 1 mg/kg prednisolone (Prednol; Mustafa Nevzat, Istanbul, Turkey), with a 10 mg taper every 3 days. The oral steroid therapy lasted about 3 weeks. In addition, the patients received lansoprazole (Lansor; Sanovel, Istanbul, Turkey) as a proton pump inhibitor for gastrointestinal protection, and they were instructed to avoid salt and high carbohydrate diets. The protocol of the HBO treatment was two sessions per day during the first 3 days, and one session per day during the following days, for a total of 20 sessions at 2.5 ATA with 120 min per session.

The clinical and audiological findings and responses to treatment were compared between the groups, as well as within the groups of patients (pre-treatment to post-treatment). The one-way ANOVA, Kruskal–Wallis, Chi squared, and Mann–Whitney *U* tests were used, and the statistical analyses were conducted with IBM SPSS software version 19 (IBM Corporation, New York, USA). The means and standard deviations were calculated, and a *p* < 0.05 was considered to be significant.

## Results

The charts of 857 patients with SHL were reviewed, and 132 patients were included in the study. The demographic data, and clinical and audiological findings are presented in Table 1. Overall, 106 (56 male, 50 female) patients presented with unilateral SHL, and 26 patients (16 male, 10 female) presented with bilateral SHL. No significant difference was observed between the groups of patients with regard to gender. The ages of the patients with unilateral SHL ranged between 12 and 58 years old, with a mean of 42.0 ± 15.0, and the ages of the patients with bilateral SHL ranged between 7 and 57 years, with a mean of 24.5 ± 19.8. There was a significant difference between the ages of the groups (*p* < 0.001) (Table 1).

**Table 1:** Demographics, clinical findings, tinnitus scores and audiologic data of the patients with unilateral and bilateral SHL (*p*-values are statistics between the groups).

	Unilateral SHL	Bilateral SHL	*p*
Number of patients	106	26	
Gender (F/M)	50/56	10/16	0.76
Av. age (mean ± st.d. [range])	42.0 ± 15.0 (12–58)	24.5 ± 19.8 (7–57)	0.001
Duration of symptoms (day [range])	4.1 ± 5.6 (0–30)	4.5 ± 3.5 (1–10)	0.68
Baseline audiogram (Av. pure tone – dB)	69.1 ± 31.1	L: 63.3 ± 18.6	0.3
		R: 67.6 ± 20.8	0.8
Post-treatment audiogram (Av. pure tone – dB)	47.0 ± 32.4	L: 55.4 ± 22.7	0.001
		R: 59.0 ± 21.7	0.001
Hearing improvement (dB)	22.1	8.2 (average of 2 ears)	0.001
Audiogram type	Type 0: 46	Type 0: 5	0.37
	Type 1: 60	Type 1: 8	
Vertigo or dizziness	(−): 78	(−): 24	0.18
	(+): 28	(+): 2	
Tinnitus	(−): 18	(−): 4	0.12
	(+): 88	(+): 22	
Pre-treatment tinnitus (Av. of THI score)	2.9 ± 1.4	2.7 ± 1.5	0.87
Post-treatment tinnitus (Av. of THI score)	1.9 ± 1.2	2.0 ± 0.8	0.12
CT and MRI finding (abnormal/normal)	0/106	0/26	–
Cardiovascular risk factors (±)	23/83	0/26	0.07
History of upper respiratory tract infection (±)	32/74	6/20	0.75

USSHL, unilateral sudden sensorineural hearing loss; BSSHL, bilateral suddan sensorineural hearing loss.

The patients with unilateral SHL presented between 0 and 30 days, with a mean of 4.1 ± 5.6, and the presentation of the patients with bilateral SHL was between 1 and 10 days, with a mean of 4.5 ± 3.5. There was no statistically significant difference in the duration of the hearing loss between the groups (*p* = 0.68). Twenty-eight of the patients (26.4%) with unilateral SHL presented with vertigo or dizziness, while only two of the patients with bilateral SHL had this type of symptom, without any statistical significance. In addition, 23 of the patients (21%) with unilateral SHL had cardiovascular risks, but none of the patients (0%) with bilateral SHL had them. The difference between the groups was very close to significance (*p* = 0.07). Moreover, in the unilateral SHL group, 32 of the patients (30%) had upper respiratory tract infections before the onset of SHL, while 6 of the patients with bilateral SHL had previous upper respiratory tract infections (23%), without statistical significance.

None of the patients with unilateral or bilateral SHL demonstrated abnormalities in their MRI or CT scans. However, 28 of the patients (26.4%) with unilateral SHL had abnormal laboratory test results: the total cholesterol levels were abnormal in 14 of the patients, the ESR was high in 2 patients, the ANA was high in 1 patient, the thyroid function tests demonstrated hypofunction in 2 patients, and the serum glucose levels were high in 9 patients. In addition, 4 of the patients (15%) with bilateral SHL had abnormal laboratory results: 2 patients had ANA positivity and 2 patients had an increased ESRs.

The pre-treatment audiological threshold of the patients with unilateral SHL was 69.1 ± 31.1 dB. In the bilateral SHL group, the baseline audiological threshold was 63.3 ± 18.6 dB for the left ears and 67.6 ± 20.8 dB for the right ears. However, there was no significant difference between the groups with regard to the baseline thresholds (Table 1). For the audiogram types, 46 patients had type 0 and 60 patients had type 1 in the unilateral SHL group. Five of the patients had type 0 and 8 had type 1 in bilateral SHL group, but there was no statistical significance between the groups (Table 1).

Tinnitus was present in 88 (83%) of the patients with unilateral SHL and 22 (84%) of the patients with bilateral SHL. The pre-treatment average tinnitus scores of the patients with unilateral and bilateral SHL were 2.9 ± 1.4 and 2.7 ± 1.5, respectively, and the difference was not significant. The post-treatment average tinnitus scores decreased to 1.9 ± 1.2 in the unilateral SHL group and 2.0 ± 0.8 in the bilateral SHL group, but again, the difference was not significant. The pre to post-treatment difference in the average tinnitus score in those patients with unilateral SHL was statistically significant (*p* = 0.01). In addition, the improvement in the average tinnitus score was also significant in the patients with bilateral SHL (*p* = 0.02) (Table 2).

**Table 2:** Audiologic thresholds and tinnitus scores of the patients in unilateral and bilateral SHL (*p* values are in-group statistics).

	Pre-treatment	Post-treatment	*p*
*USSHL*			
Audiologic thresholds (Av. pure tone – dB)	69.1 ± 31.1	47.0 ± 32.4	0.001
Tinnitus scores (Av. of THI score)	2.9 ± 1.4	1.9 ± 1.2	0.01

*BSSHL*			
Audiologic thresholds – left (Av. pure tone – dB)	63.3 ± 18.6	55.4 ± 22.7	0.56
Audiologic thresholds – right (Av. pure tone – dB)	67.6 ± 20.8	59.0 ± 21.7	0.09
Tinnitus scores (Av. of THI score)	2.7 ± 1.5	2.0 ± 0.8	0.02

USSHL, unilateral sudden sensorineural hearing loss; BSSHL, bilateral suddan sensorineural hearing loss.

The post-treatment average hearing threshold of the patients with unilateral SHL was 47.0 ± 32.5 dB. The corresponding hearing thresholds of the bilateral SHL patients were 46.0 ± 22.7 dB for the left ears and 59.0 ± 21.7 dB for the right ears. While the average hearing improvement was 22.1 dB in the unilateral SHL group, it was 7.8 dB for the left ears, 8.6 dB for the right ears and 8.2 dB for the average of the two ears in the bilateral SHL group. There was a significant difference between the groups according to the average hearing improvement (*p* < 0.001) (Table 1).

Overall, there was a significant improvement with treatment on the hearing in the unilateral SHL group (*p* < 0.001). However, the hearing improvements of both ears in the bilateral SHL group were not statistically significant (*p* = 0.56 for the left ears and *p* = 0.09 for the right ears) (Table 2).

## Discussion

SHL is a disorder affecting adults that is not uncommon, which causes a severe limitation in the quality of life, and urgent management is necessary to improve hearing recovery.^10^, ^11^ SHL is defined as a rapid onset (occurring over a 72 h period) sensorineural type of hearing impairment in one or both ears. The most frequently used audiological criterion is a decrease in the hearing threshold of more than 30 dB in 3 consecutive frequencies.^11^

Most patients with SHL present with a unilateral disorder, and bilateral involvement is very rare.^4^ There have been some case reports describing SHL in both ears in patients with bilateral temporal bone fractures,^12^ temporal intracerebral haemorrhage,^13^ meningeal carcinomatosis,^14^ metastatic tumours,^15^ or drug use.^16^ To define the sudden hearing loss as idiopathic, no identifiable cause must be found, despite adequate investigation. In our series, the patients were evaluated with a detailed history, including drug use, and CT and MRI examinations to identify an underlying cause. None of the patients with SHL demonstrated any disorders related to hearing loss.

In this study, the ages of the patients with unilateral SHL were significantly higher than those of the patients with bilateral SHL (42.0 ± 15.0 vs. 24.5 ± 19.8, respectively). Although the mean age of the patients with bilateral SHL was higher than that of the unilateral group in the common literature,^4^, ^10^ it is not possible to speculate that bilateral SHL is more frequently seen in the younger age group, according to our results. Attention should be drawn to this contradiction.

In some studies of SHL, bilateral involvement ranges from 0.44% to 4.9% of the patients.^1^, ^2^, ^3^, ^4^, ^5^ In their study, Kiriake et al. reported 7 patients out of 205 cases of SHL.^17^ Fetterman et al. reported 1.7% bilateral involvement in 823 patients, while Oh et al. reported 4.9% in 324 patients.^10^ In our study, we evaluated 857 patients with SHL, and detected simultaneous bilateral ear involvement in 26 patients (3%), which was similar to the current literature. We included cases that were all given the same treatment, and a total of 132 patients were appropriate for the analysis.

There has been some speculation with regard to the cause of SHL, including viral infection, vascular insufficiency, rupture of the labyrinthine membranes and autoimmune reactions.^3^, ^18^ As in the unilateral cases, those disorders that lead to bilateral SHL are also thought to be vascular, metabolic, traumatic, autoimmune, infectious, toxic, neoplastic or inflammatory.^11^ There have been some reports supporting a viral aetiology in the development of bilateral SHL.^19^ For example, Fetterman et al. reported that the incidences of previous viral illness were higher in the cases of bilateral SHL, but without any statistical significance.^4^ However, Xenellis et al. detected a history of viral disease in only 9% of their patients with bilateral SHL.^20^ In the study by Oh et al., there was no difference between the unilateral and bilateral SHL cases with regard to a history of viral disease.^10^ In this study, 32 (30%) of the patients with unilateral SHL and 6 (23%) of the patients with bilateral SHL had previous viral diseases before the onset of hearing loss. Although the incidence was higher in the unilateral group, the difference was not significant. We do not believe that viral infection is an effective etiological agent to show a difference between the groups.

The vascular insufficiency theory was supported in the report by Fetterman et al.^4^ They found that those patients with bilateral SHL had a 3 fold higher incidence of cardiovascular disease than the patients with unilateral SHL. Oh et al. detected a significantly higher incidence of systemic cardiovascular disorders, but concluded that peripheral circular dysfunction could not be the major mechanism for the development of bilateral SHL.^10^ Conversely, Xenellis et al. could not find any cardiovascular diseases in their patients with bilateral SHL,^20^ which was supported by our study. According to our results, cardiovascular risk factors seemed to play an important role in the aetiology of the unilateral cases, but this importance was not present in the bilateral ones. Oh et al. found significantly increased lipid levels in their patients with bilateral SHL, which might constitute a risk factor.^10^ The most frequently encountered abnormal laboratory finding in this study was hyperlipidaemia, which was only detected in the patients with unilateral SHL. This could be another supportive result for the opinion about cardiovascular risks stated above.

In our study, we detected an increased ANA level in only one patient, and an increased ESR in two patients from the unilateral SHL group. Two of the patients in the bilateral SHL group presented with ANA positivity, while two other patients had increased ESRs. The abnormal laboratory findings were evaluated together, and although the differences between the groups were insignificant, the patients with bilateral SHL had a tendency towards higher levels of immune response markers (3/106 patients in the unilateral SHL group and 4/26 patients in the bilateral SHL group).

Overall, SHL treatment remains controversial. While some authors believe that steroid treatment is no better than a placebo,^21^ some meta-analyses have shown a slight, but not statistically significant, benefit from medical treatment over a placebo.^22^ Although the data do not strongly support its use, corticosteroid treatment is one option that demonstrates efficiency.^11^ We routinely prescribe oral steroids to suppress the inflammation and possible autoimmunity within the cochlea. The use of HBO is one method for the treatment of SHL, and there have been some reports demonstrating the beneficial effects of HBO in SHL by delivering oxygen to the cochlea with increased partial pressure.^11^ In the recent guidelines published by Stachler et al., clinicians can offer HBO treatment within 3 months of the diagnosis of SHL.^11^ We work with a highly experienced HBO therapy centre, and have routinely used HBO therapy combined with oral steroids since the year 2000. We believe that increasing the partial pressure of the oxygen increases the oxygenation of the injured tissues, and improves the healing process.

There is some conflicting data about the recovery from bilateral SHL in the current literature. For instance, the study by Xenelis et al. stated that the improvement was higher in the cases of unilateral SHL than in bilateral SHL,^20^ while Fetterman et al. reported hearing improvement in 67% of their patients with bilateral SHL, which was not significantly different from the unilateral cases.^4^ Our findings demonstrated that the hearing thresholds of both groups improved with the steroid plus HBO treatments, but the hearing improvement was more pronounced in unilateral SHL group.

Tinnitus is a very common co-existing condition in SHL that is very hard to treat, and it creates significant economic and psychological problems.^23^ Nearly 90% of the patients with SHL suffer from tinnitus, which is a positive prognostic factor for hearing recovery, but a longer duration of tinnitus predicts poor results.^24^ In previous studies about bilateral SHL, the percentage of tinnitus was around 80%, which was not different from the results from our study.^4^, ^20^ The patients in this study were also evaluated using the THI, in which both groups’ tinnitus scores regressed significantly, and the final scores after treatment between the groups were not significantly different. This may show the effects of the steroid HBO combination treatment on the regression of tinnitus.

The recovery from sudden hearing loss depends on certain factors, like a patient's age, duration of symptoms, the severity and pattern of the hearing loss, and the presence of vestibular symptoms.^3^, ^10^, ^25^ The mean age of the patients with bilateral SHL represented a better prognostic factor in this study. Only two patients (7.6%) in the bilateral SHL group and 28 patients (26%) in the unilateral SHL group had vertigo or dizziness, but this was not a significant difference. The tinnitus scores and audiogram patterns of the two groups also showed no significant differences. In light of these facts, we can say that age was the only effective prognostic factor between the groups. In spite of this, the prognosis was worse in the bilateral SHL group.

## Conclusion

Bilateral SHL exhibits different clinical features than unilateral SHL. Overall, our study demonstrated some opposing findings about bilateral SHL when compared to the current literature. For example, the mean age of our patients was younger, which showed that bilateral SHL was not a disease of older ages. Moreover, those patients with bilateral SHL demonstrated poorer prognoses, a higher incidence of immune response markers and a lower incidence of cardiovascular risk factors.

The studies about bilateral SHL in the current literature are insufficient when considering the sample sizes. Therefore, we believe that it is not possible to conclude definite results, but bilateral SHL seems to be the result of a different process than in the unilateral cases, and should be seriously considered.

## Ethical approval

All procedures performed in studies involving human participants were in accordance with the ethical standards of the institutional and/or national research.

## Conflicts of interest

The authors declare no conflicts of interest.
